# Small nucleolar RNAs as new biomarkers in chronic lymphocytic leukemia

**DOI:** 10.1186/1755-8794-6-27

**Published:** 2013-09-03

**Authors:** Domenica Ronchetti, Laura Mosca, Giovanna Cutrona, Giacomo Tuana, Massimo Gentile, Sonia Fabris, Luca Agnelli, Gabriella Ciceri, Serena Matis, Carlotta Massucco, Monica Colombo, Daniele Reverberi, Anna Grazia Recchia, Sabrina Bossio, Massimo Negrini, Pierfrancesco Tassone, Fortunato Morabito, Manlio Ferrarini, Antonino Neri

**Affiliations:** 1 Department of Clinical Sciences and Community Health, University of Milano, Milano, Italy; 2 SS Molecular Diagnostics, IRCCS S, Martino-IST, Genova, Italy; 3 Department of Clinical Sciences and Community Health, University of Milan, Hematology 1 CTMO, Fondazione IRCCS Ca’ Granda Ospedale Maggiore Policlinico, F. Sforza, 35-20122, Milano, Italy; 4 U.O.C. di Ematologia, Azienda Ospedaliera di Cosenza, Cosenza, Italy; 5 Scientific Direction, IRCCS S Martino-IST, Genova, Italy; 6 Department of Pathology IRCCS S Martino-IST, Genova, Italy; 7 Department of Experimental and Clinical Medicine, University of Ferrara, Ferrara, Italy; 8 Department of Experimental and Clinical Medicine, Magna Graecia University, Catanzaro, Italy

## Abstract

**Background:**

Small nucleolar RNAs (snoRNAs) and small Cajal body-specific RNAs are non-coding RNAs involved in the maturation of other RNA molecules. Alterations of sno/scaRNA expression may play a role in cancerogenesis. This study elucidates the patterns of sno/scaRNA expression in 211 chronic lymphocytic leukemia (CLL) patients (Binet stage A) also in comparison with those of different normal B-cell subsets.

**Methods:**

The patterns of sno/scaRNA expression in highly purified CD19^+^ B-cells of 211 CLL patients and in 18 normal B-cell samples - 6 from peripheral blood, and 12 from tonsils (4 germinal center, 2 marginal zone, 3 switched memory and 3 naïve B-cells) - were analyzed on the Affymetrix GeneChip® Human Gene 1.0 ST array.

**Results:**

CLLs display a sno/scaRNAs expression profile similar to normal memory, naïve and marginal-zone B-cells, with the exception of a few down-regulated transcripts (SNORA31, -6, -62, and -71C). Our analyses also suggest some heterogeneity in the pattern of sno/scaRNAs expression which is apparently unrelated to the major biological (ZAP-70 and CD38), molecular (*IGHV* mutation) and cytogenetic markers. Moreover, we found that SNORA70F was significantly down-regulated in poor prognostic subgroups and this phenomenon was associated with the down-regulation of its host gene *COBLL1*. Finally, we generated an independent model based on SNORA74A and SNORD116-18 expression, which appears to distinguish two different prognostic CLL groups.

**Conclusions:**

These data extend the view of sno/scaRNAs deregulation in cancer and may contribute to discover novel biomarkers associated with the disease and potentially useful to predict the clinical outcome of early stage CLL patients.

## Background

Chronic lymphocytic leukemia (CLL) is a clinically heterogeneous disease. Some patients have an indolent course and may survive for years without treatment, while others have an aggressive and rapidly progressive course [1,2]. Although the current Rai [3] and Binet [4] staging systems identify patients with high-risk disease, they do not prospectively distinguish patients with potentially evolving disease from those destined to remain stable for decades.

A number of cellular and molecular markers help to classify CLL into biologically and clinically distinct subgroups, and to predict the clinical course of the disease at diagnosis [5]. CLL patients with unmutated (UM-CLL) immunoglobulin heavy chain variable (*IGHV*) region genes (>98% homology to germline sequences), increased expression of the CD38 cell surface antigen, or of the 70-kd zeta-chain T-cell receptor–associated protein kinase (ZAP-70) experience a shorter therapy-free interval, a more aggressive clinical course, and a shorter survival [6-8]. Specific recurrent chromosomal abnormalities, traditionally detected by Fluorescence *in situ* Hybridization (FISH), such as deletions at 13q14 (del13), 11q23 (del11), 17p13 (del17), and trisomy 12 (12+) may also represent important independent biomarkers for disease progression and survival [9-13].

In recent years, the discovery of non-coding RNAs (ncRNAs) has provided new tools for the understanding of cancer biology [14]. ncRNAs are functional transcripts that do not code for proteins, but indeed play a major role in regulating gene expression at virtually all levels. Among these, small nucleolar RNAs (snoRNAs) are molecules of 60–300 nucleotides in length that function as guide RNAs for the post-transcriptional methylation and/or pseudouridylation of ribosomal RNAs. Unlike snoRNAs, small Cajal body-specific RNAs (scaRNAs) accumulate within the Cajal bodies (i.e. conserved subnuclear organelles that are present in the nucleoplasm) and can guide both post-transcriptional modifications of small nuclear RNAs. sno/scaRNAs can also target other RNAs including mRNAs. However, a subgroup of so-called “orphan” sno/scaRNAs exists whose function remain to be clarified. Notably, in vertebrates most sno/scaRNAs reside in the introns of protein-coding host genes and are processed out of the excised introns [15].

Experimental evidence has recently shown that sno/scaRNAs dysfunction may have a role in the origin of human cancers [16]. Limited data concerning the involvement of sno/scaRNAs in hematological malignancies have been reported only very recently by us and others [17-22], and are lacking in CLL.

In the present study, we investigated the sno/scaRNA expression profiling in a large and representative prospective series of Binet stage A CLL patients. In addition, we evaluated sno/scaRNAs expression in comparison to that observed in various sub-populations of normal B-cells from tonsils and peripheral blood B-lymphocytes. Finally, the sno/scaRNA expression pattern was correlated with clinical data in order to outline their possible role in CLL prognosis.

## Methods

### Patients

Two hundred and eleven newly diagnosed CLL patients were prospectively enrolled from several Italian Institutions in an observational multicenter study (O-CLL1 protocol, clinical trial.gov identifier NCT00917540) from January 2007 to May 2011. The National Cancer Institute (NCI)-sponsored Working Group guidelines were followed for diagnosis and staging [23]. All patients had an absolute lymphocyte count (ALC) greater than 5000×10^9^/L in the peripheral blood. Exclusion criteria were the following: i) a CLL diagnosis exceeding 12 months before registration; ii) a leukemic phase of lymphoproliferative disorders of B origin, CD5^-^ and/or CD23^-^ according to flow cytometry analysis; iii) a clinical Binet stage B or C; iv) need of therapy according to NCI guidelines and v) age > 70 years. The median follow-up of this series was 30 months. Diagnosis was confirmed by the biological review committee according to flow cytometry analysis centralized at the National Cancer Institute (IST) Laboratory in Genoa. Written informed consent was obtained from all patients in accordance with the declaration of Helsinki and the study was approved by the local Ethics Review Committee (Comitato Etico Provinciale, Modena, Italy).

Peripheral blood mononuclear cells from CLL patients and normal control, and B-cell sub-populations from tonsils (i.e. naïve B-cells (N), marginal zone (MZ)-like, germinal center (GC) and switched memory (SM) B-cells) were obtained as described in Additional file 1 and Additional file 2.

Biological and molecular analyses were performed in highly enriched CD19^+^ B-cells as previously described [24,25]. CD38 and ZAP-70 expressions were investigated using a cut-off of 20% and 30% respectively to discriminate positive from negative patients. Del11, del13, del17, and 12+, *IGHV* mutation status and stereotyped BCR subtypes were determined as previously described [26-28]. Specifically, *IGHV* sequences from 210 patients were submitted to IMGT V-QUEST analysis software, and were classified as mutated (M-CLL) or UM-CLL using a discriminating mutation cut-off percentage at 2%.

### Gene expression profiling (GEP)

Total RNA from purified CD19^+^ B-cells of 211 CLL cases and 18 normal B-cell samples - 6 from peripheral blood, and 12 from tonsils (4 germinal center, 2 marginal zone, 3 switched memory and 3 naïve B-cells) - were analyzed on the GeneChip® Human Gene 1.0 ST array (Affymetrix Inc., Santa Clara, CA). The raw intensity expression values were processed by Robust Multi-array Average (RMA) procedure [29] with the re-annotated Chip Definition Files (CDF) from BrainArray libraries version 15.0.0 [30] available at http://brainarray.mbni.med.umich.edu. This custom CDF reorganized probe sets based on i) more stringent gene/transcript definitions from UniGene database, and ii) alignment analyses performed on each single probe included in the probeset [30]. The updated annotations provided a better precision and accuracy in transcript expression level estimates compared to the original Affymetrix definitions [19,31].

Supervised analyses were carried out using the Significant Analysis of Microarrays software version 4.00 (SAM; excel front-end publicly available at http://www-stat.stanford.edu/~tibs/SAM/index.html) [32]. The cutoff point for statistical significance (at a *q*-value 0) was determined by tuning the Δ parameter on the false discovery rate and controlling the *q*-value of the selected probes. Hierarchical agglomerative clustering of the most significant probesets found was performed adopting Pearson and average as distance and linkage methods, respectively. DNA-Chip Analyzer software (dChip) [33] was used to perform clustering and to graphically represent it. GEP data have been deposited in the NCBI Gene Expression Omnibus database (GEO; http://www.ncbi.nlm.nih.gov/geo/) under accession #GSE46261.

In order to explore the distribution of CLL samples we applied principal component analysis (PCA) on sno/scaRNA microarray expression data. PCA was performed by means of the *princomp* function in R software, by scaling data and using the associated correlation matrix. Hence we used the *3dplot* to visualize samples by the first three principal components.

### Statistical analysis

We used the Cox proportional hazards model in the *globaltest* function of R software (with 100,000 permutation) to test the association (positive or negative) between sno/scaRNA expression levels, assumed as continuous variables and progression free survival (PFS) as clinical outcome. The list of significant associated sno/scaRNA to the PFS was reduced to derive a smaller prognostic sno/scaRNA signature by employing single sno/scaRNA expression profile. Unsupervised K-means clustering was applied to each sno/scaRNA to define a threshold splitting samples with higher expression from those with lower expression (Additional file 3), in order to test their different clinical outcome. The survival distributions of patient groups identified by this approach were tested using the Kaplan-Meier estimator and log-rank test, and P-values were calculated according to the standard normal asymptotic distribution (*survdiff* function of the *survival* R package). The Benjamini-Hochberg correction has been applied to consider the false discovery rate that incurred in the multiple testing. Independence between commonly used CLL prognostic factors (*IGHV* mutational status, ZAP-70, CD38 and unfavorable chromosome aberrations, namely del11, del17 and 12+) and sno/scaRNAs signature was assessed using multivariate Cox proportional-hazards regression procedure by the *coxph* function in the *survival* R package. The robustness of the 2-snoRNA model was tested using the linear discriminant analysis for the classification of multivariate observations, with leave-one-out procedure, using *lda*function in *MASS* R package. Other statistical procedures were applied using standard functions in *base* R package (Kendall Tau correlations and Wilcoxon rank-sum tests).

## Results

### Characteristics of CLL patients

Two hundred eleven CLL patients at Binet stage A were included in the study; most of them were Rai 0 stage (74.4%). The median follow-up was 30 months (range 1–65 months). CLL cell samples were characterized for *IGHV* somatic mutations, ZAP-70 and CD38 expression and the presence of typical chromosomal aberrations (del13, del11, del17, and 12+) detected by FISH analysis. Table 1 summarizes the features of CLL samples.

**Table 1:** Characteristics of CLL patients cohort

	**CLL (% of total)**
**N° of patients**	**211**
**Rai stage**	
0	157 (74.4)
I	40 (19)
II	14 (6.6)
**CD38**	
Negative	148 (70.1)
Positive	63 (29.9)
**ZAP-70**	
Negative	115 (54,5)
Positive	96 (45.5)
***IGHV***	
M-CLL	127 (60.2)
UM-CLL	83 (39.3)
nd	1 (0.5)
**FISH**	
No abnormality	76 (36)
del13	102 (48.3)
12+	28 (13.2)
del11	16 (7.6)
del17	5 (2.4)

### sno/scaRNA expression profile of CLL cells compared to normal B-cell populations

The expression profiles of sno/scaRNA genes were investigated using Human Gene 1.0 ST arrays. Oligonucleotide probes were mapped according to the genome and transcriptome information from the human genome build release GRCh37/hg19 (see Methods) identifying 215 human snoRNA (76 SNORA and 139 SNORD transcripts) and 17 human scaRNA genes. The expression patterns also were studied in normal B-cells from peripheral blood (pBC) and in different tonsil B-cell sub-populations including germinal center (GC), naïve (N), marginal zone (MZ) and memory (SM) B-cells (Additional file 2 reports the FACS sorting strategy used to purify these cell subpopulations as described in Additional file 1).

**Figure 1:**
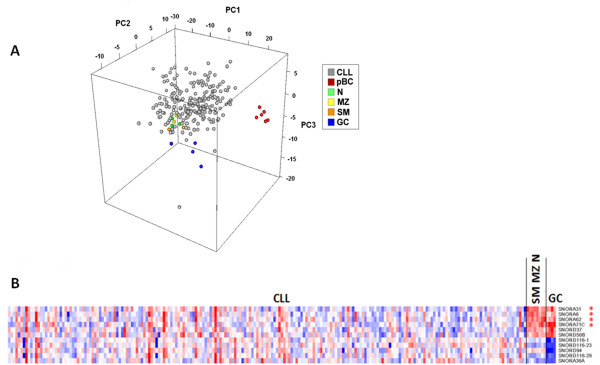
**sno/scaRNA expression profile of CLL and normal B-cells.** A PCA analysis that includes CLL samples shows that CLLs are closer in a three-dimensional space of similarity to SM, N and MZ tonsillar B-lymphocytes than to other B-cell types, based on the expression of 215 snoRNA and 17 scaRNA genes **(A)**. Heatmap of the differentially expressed sno/scaRNAs in CLL patients, N-SM-MZ B-cells, and GC B-cells. snoRNAs also resulting from two-class supervised analysis comparing CLL and N-SM-MZ B-cells group, are marked with an asterisk **(B)**.

At first, we applied PCA on the sno/scaRNA dataset composed by 211 CLL and 18 normal cell samples; the first three PCs contained 51% of variance in our data and we used them to display all the cell type samples in a three-dimensional plot. As shown in Figure 1A, we observed that CLL, N, MZ and SM B-cell samples were clustered together, whereas GC and in particular pBC samples, were well-separated from other groups along PC3 and PC1 components, respectively. Based on this evidence, multiclass analysis was performed comparing CLLs with N, MZ and SM (taken as a single group) and GC B-cells. Despite the heterogeneity of snoRNAs expression in CLL samples, we confirmed that the sno/scaRNA expression profile of CLL was much more similar to N-MZ-SM B-cells rather than to GC (Figure 1A-B). Specifically, we observed the significant down-regulation of SNORA31, -6, -62, and 71C, SNORD37, and -50B in CLL with respect to tonsillar B-cells; however, GC B-cells differed from both CLL and N-MZ-SM B-cells on the basis of a number of down-regulated transcripts, such as SNORD116-1, -116-23, -116-29, -94, and SNORA36A (Figure 1B).

Notably, SNORA31, -6, -62, and -71C also resulted significantly down-regulated in each of the CLL subgroups stratified according to genetic lesions (del13, del11, del17, 12+ and FISH negative) in a two-class supervised analysis with the N, MZ and SM group (data not shown). Finally, in agreement with PCA, supervised analysis confirmed the strong differences in sno/scaRNA expression between pBC and CLL samples, showing the up-regulation of 102 sno/scaRNAs and the down-regulation of a single transcript (SNORA36C) in CLL patients (Additional file 4).

### Global sno/scaRNA expression profiling in CLL patients

To determine whether the natural grouping of global sno/scaRNA expression profiling could be associated with particular CLL subgroups, we performed an unsupervised analysis using conventional hierarchical agglomerative clustering of 71 snoRNAs and 9 scaRNAs, the average change of which varied at least 1.5-fold in the expression levels from the mean values across the dataset (Additional file 5 and Additional file 6). However, CLL samples appeared scattered along the dendrogram irrespective of any known molecular characteristic.

Consequently, we performed supervised analyses (*q* value = 0) to investigate the occurrence of specific sno/scaRNA expression patterns in CLL groups stratified according to the presence/absence of different prognostic markers; i.e. *IGHV* gene mutations and ZAP-70 and CD38 expression. Significant differences were detected only in the expression of a limited number of transcripts in the groups stratified as above (Table 2). Specifically, UM-CLLs showed the down-regulation of SNORA70F and SNORA70C, and the up-regulation of SNORA71C. Of note, SNORA70F also was found down-regulated in ZAP-70 positive and in CD38 positive CLL patients, in whom SNORA70C was also down-regulated. Furthermore, snoRNAs expression was investigated in cases stratified according to the presence of chromosomal alterations such as 12+, del11, del13, and del17 by supervised analyses (at *q* value = 0). SNORA70F was significantly down-regulated in CLL patients with 12+ or del11 (Table 2). Overall, lower expression levels of SNORA70F were associated with the cumulative adverse biological and molecular features (see Additional file 7). Moreover, 12+ patients displayed the significant down-regulation of SCARNA17 and up-regulation of three snoRNAs located on chromosome 12 (SNORA2B, SNORD59A, and -59B) (Table 2). The significant down-regulation of SCARNA9 which is located at 11q21 (Table 2), was also observed in cases with del11. Interestingly, in 8/13 del11 cases, for which genome-wide data were available, we observed that the SCARNA9 locus was included in the deleted region (data not shown). By comparing patients with/without del13 we identified 17 snoRNAs and 2 scaRNAs consistently down-regulated in del13 samples (Table 2). Finally, supervised analysis of CLL samples with del17 did not show any differentially expressed sno/scaRNAs, a finding that could be, however, related to the low number of cases.

**Table 2:** **Supervised analysis comparing CLLs with adverse *vs *favorable prognostic factors**

**NEGATIVE PROGNOSTIC FACTOR**	**sno/scaRNA**	**Alias**	**Cytoband**	**Score(d)**	**Fold change**
**IGHV unmutated**	**SNORA70F**	U70F	2q24	−10.81	0.30
	**SNORA70C**	U70C	9q33	−3.58	0.87
	**SNORA71C**	U71C	20q11	3.54	1.23
**ZAP70 pos**	**SNORA70F**	U70F	2q24	−6.91	0.43
**CD38 pos**	**SNORA70F**	U70F	2q24	−6.67	0.41
	**SNORA70C**	U70C	9q33	−3.46	0.86
**12+**	**SNORA70F**	U70F	2q24	−3.88	0.46
	**SCARNA17**	mgU12-22/U4-8	18q21.1	−3.58	0.72
	**SNORD59A**	U59	12q13.3	3.58	1.38
	**SNORA2B**	ACA2B	12q13.11	3.69	1.40
	**SNORD59B**	U59B	12q13.3	3.89	1.43
**del11**	**SNORA70F**	U70F	2q24	−4.00	0.37
	**SCARNA9**	mgU2-19/30	11q21	−3.75	0.62
**del13**	**SNORA16A**	ACA16	1p35	−5.24	0.62
	**SNORA70G**	U70G	12q15	−4.98	0.80
	**SNORA24**	ACA24	4q26	−4.87	0.65
	**SNORD38A**	U38A	1p34	−4.53	0.77
	**SNORA20**	ACA20	6q25	−4.34	0.74
	**SNORD60**	U60	16p13	−4.34	0.63
	**SNORD54**	U54	8q12	−4.31	0.73
	**SNORD50A**	U50	6q14	−4.28	0.70
	**SNORD45B**	U45B	1p31	−4.21	0.57
	**SNORD76**	U76	1q25	−4.20	0.75
	**SNORD34**	U34	19q13	−4.14	0.75
	**SCARNA7**	U90	3q25	−4.08	0.79
	**SNORD8**	mgU6-53	14q11	−4.06	0.74
	**SNORD15B**	U15B	11q13	−4.02	0.73
	**SNORD38B**	U38B	1p34	−4.00	0.69
	**SNORD59B**	U59B	12q13	−3.98	0.78
	**SNORD104**	U104	17q23	−3.88	0.77
	**SNORA68**	U68	19p13	−3.76	0.79
	**SCARNA10**	U85	12p13	−3.68	0.76

*Abbreviations:scaRNA* small Cajal body-specific RNA, *snoRNA* small nucleolar RNA.

### Correlation of sno/scaRNAs expression levels with those of their corresponding host genes

The vast majority of human sno/scaRNAs are encoded sense-oriented in introns of so-called host genes, and are produced by exonucleolytic processing of the debranched intron after splicing. This suggests that sno/scaRNAs and their host genes may have a coordinate expression. Among 169 sno/scaRNA-host gene couples present on Human Gene 1.0 ST array, we identified by means of the non parametric Kendall τ correlation test, 75 snoRNAs and 4 scaRNAs whose expression levels were significantly correlated with those of their corresponding host genes (Additional file 8); this list includes 15 sno/scaRNAs resulting from the supervised analyses carried out in our study (Table 3). Specifically, SNORA2B and the SNORD59A, -59B cluster, all of which located at 12q13, were up-regulated in patients with 12+ together with their corresponding host transcripts c12orf41 and *ATP5B* (mitochondrial ATP synthase). Similarly, the expression of SNORA31, which is located at 13q14, was correlated with that of the host gene *TPT1*. Notably, the expression of SNORA70F was significantly associated with that of its host gene *COBLL1*, whereas the expression of the SNORA6 and −62 cluster was significantly correlated with that of its host gene *RPSA*.

**Table 3:** Correlation of expression levels of sno/scaRNAs resulting from multiclass or supervised analyses and corresponding host-genes

**Sno/scaRNA**	**Alias**	**Cytoband**	**Host gene**	**Kendall *q*-value**	**Target RNA**
**SNORD45B**	**U45B**	**1p31.1**	**RABGGTB**	**7.260E-14**	**18S rRNA A159 and 18S rRNA U172**
**SNORD38A**	U38A	1p34	RPS8	A^a^	28S rRNA A1858
**SNORD38B**	U38B	1p34	RPS8	A^a^	28S rRNA A1858
**SNORA16A**	**ACA16**	**1p35.3**	**SNHG12**	**2.540E-02**	**28S rRNA U4412**
**SNORD76**	U76	1q25	GAS5	A^a^	28S rRNA A2350
**SNORD94**	U94	2p11.2	PTCD3	5.609E-01	U6 snRNA C62
**SNORA70F**	**U70F**	**2q24**	**COBLL1**	**<2.2e-16**	**18S rRNA U1692**
**SNORA6**	**ACA6**	**3p22**	**RPSA**	**4.180E-06**	**28S rRNA U3616**
**SNORA62**	**E2**	**3p22**	**RPSA**	**6.380E-06**	**28S rRNA U3830 and 28S rRNA U3832**
**SCARNA7**	U90	3q25	KPNA4	8.912E-01	U1 snRNA A70
**SNORA24**	ACA24	4q26	SNHG8	A^a^	18S rRNA U863 and 18S rRNA U609
**SNORD50A**	U50	6q14	SNHG5	A^a^	28S rRNA C2848 and 28S rRNA G2863
**SNORA20**	ACA20	6q25	TCP1	1.000E+00	18S rRNA U651
**SNORD54**	**U54**	**8q12.1**	**RPS20**	**<2.2e-16**	**18S rRNA G644**
**SNORA70C**	**U70C**	**9q33.1**	**ASTN2**	**3.760E-06**	**18S rRNA U1692**
**SNORD15B**	**U15B**	**11q13.4**	**RPS3**	**<2.2e-16**	**28S rRNA A3764**
**SCARNA9**	mgU2-19/30	11q21	KIAA1731	1.050E-01	U2 snRNA G19 and U2 snRNA A30
**SCARNA10**	U85	12p13	NCAPD2	3.746E-01	U5 snRNA U46 and U5 snRNA C45
**SNORA2B**	**ACA2B**	**12q13.11**	**C12orf41**	**<2.2e-16**	**28S rRNA U4263 and 28S rRNA U4282**
**SNORD59B**	**U59B**	**12q13.3**	**ATP5B**	**3.530E-07**	**18S rRNA A1031**
**SNORD59A**	**U59**	**12q13.3**	**ATP5B**	**9.032E-04**	**18S rRNA A1031**
**SNORA70G**	U70G	12q15	RAB1B	1.000E+00	18S rRNA U1692
**SNORA31**	**ACA31**	**13q14.13**	**TPT1**	**2.951E-02**	**18S rRNA U218 and 28S rRNA U3713**
**SNORD8**	mgU6-53	14q11	CHD8	6.937E-01	U6 snRNA A53
**SNORD116-23**	**HBII-85-23**	**15q11.2**	**SNRPN**	**3.017E-02**	**unknown**
**SNORD116-1**	HBII-85-1	15q11.2	SNRPN	1.364E-01	unknown
**SNORD116-29**	HBII-85-29	15q11.2	SNRPN	8.127E-01	unknown
**SNORD60**	U60	16p13	LOC100507303	A^a^	28S rRNA G4340
**SNORD104**	U104	17q23	ORGUL028719	A^a^	28S rRNA C1327
**SCARNA17**	mgU12-22/U4-8	18q21.1	I^b^		U4 snRNA C8 and U12 snRNA G22
**SNORA68**	U68	19p13	RPL18A	A^a^	28S rRNA U4393
**SNORD37**	**U37**	**19p13.3**	**EEF2**	**1.845E-03**	**28S rRNA A3697**
**SNORD34**	U34	19q13	RPL13A	A^a^	28S rRNA U2824
**SNORA71C**	U71C	20q11.23	LOC388796	1.910E-01	18S rRNA U406
**SNORA36A**	**ACA36**	**Xq28**	**DKC1**	**1.561E-03**	**18S rRNA U1244 and 18S rRNA U105**

*Abbreviations:scaRNA* small Cajal body-specific RNA, *snoRNA* small nucleolar RNA. ^a^probe not present onto Human Gene 1.0 ST arrays; ^b^Independent transcriptional unit; snoRNA significantly correlating with corresponding host gene is typed in bold.

### Identification of sno/scaRNA transcriptional profile with clinical relevance in the definition of high-risk CLLs

The sno/scaRNAs transcriptional profiles were examined in relation to CLL clinical course in 191/211 CLL patients for whom clinical follow-up was almost 6 months. In particular, we evaluated whether a specific sno/scaRNA signature was significantly associated with PFS in our prospective series. To this aim, we determined possible associations between PFS and each of the 80 most variable sno/scaRNAs across the CLL dataset (*i.e.* sno/scaRNAs whose average ratio of the expression was at least 1.5 folds above the mean values). Of these 80 sno/scaRNAs, 5 showed significant association with PFS (P < .05, see Additional file 5), i.e. SNORD116-18, SNORA70F, SNORA74A, SNORD56, and SNORD1A.

As a further validation, the expression levels of SNORD1A, and SNORA74A were evaluated in 50 samples by quantitative real time PCR (qRT-PCR) as previously described [19]: the linear correlation analysis indicated a good correspondence between the two techniques (Pearson’s correlation coefficient of 0.71 and 0.76, respectively; data not shown).

**Figure 2:**
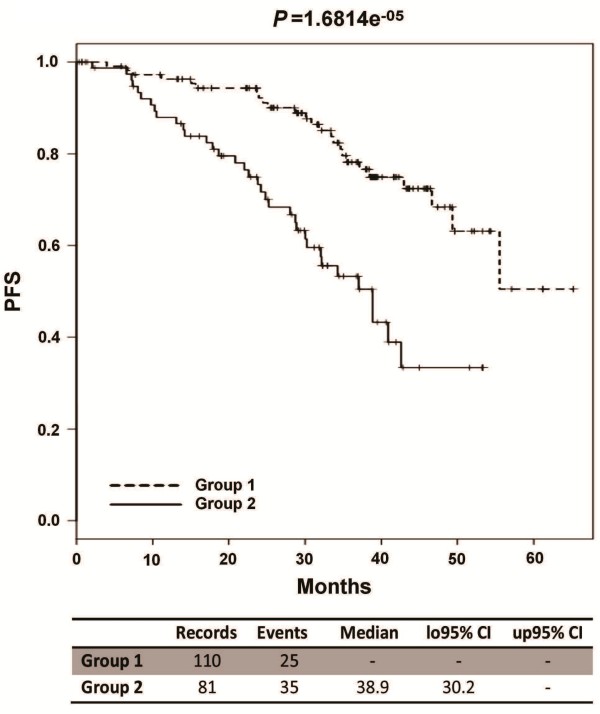
**PFS of patients grouped according to the snoRNAs-based model.** Kaplan-Meier curves of the 2 groups defined by the 2-snoRNAs model identifying a low-risk group characterized by the low expression of both snoRNAs and a high risk group characterized by the high expression of at least one of the two snoRNAs. The high risk group has a median PFS of 39 months.

Therefore, we focused on each of the 5 snoRNAs as unique predictive factor. Based on the K-means clustering stratification of CLL cases into two groups according to snoRNAs expression level, we found that each snoRNA resulted capable of providing significant predictive value related to PFS in the univariate log-rank test (P < .05, Table 4). However, in multivariate analysis, only SNORA74A, SNORD116-18 and SNORD56 retained their independence (Additional file 9). Specifically, high expression of SNORA74A and SNORD116-18, and low expression of SNORD56 were associated with shorter PFS. We therefore focused on SNORA74A and SNORD116-18, whose ratio between median high- and low-expression levels exceeded two-fold (Additional file 3), to evaluate whether their combination could improve the robustness of the single-snoRNA model, based on the scheme of three groups defined by (i) high/high, (ii) low/low or (iii) discordant expression levels. Significantly, better PFS was identified in patients with the concomitant low expression of SNORA74A and SNORD116-18, whereas no difference emerged between the two other groups, namely those including patients with high expression of both or one of the two snoRNAs (data not shown). Based on these findings, we could define a low-risk group that included 110 patients (58%) characterized by the low expression of both snoRNAs, and a high-risk group (81 patients) characterized by the high expression of at least one of the two snoRNAs. The high-risk group had a hazard ratio of 2.53 (95% CI: 1.48-4.32) and a median PFS of 39 months (Table 5 and Figure 2), versus a median PFS not reached in low-risk group. The robustness of the 2-snoRNA model was tested by leave-one-out cross validation procedure, which ultimately led to near 90% accuracy.

**Table 4:** SnoRNAs with significant predictive value related to PFS in the univariate log-rank test

**Variable**	**HR**	**Lo 95% CI**	**Up 95% CI**	***p*-val**
**SNORD1A**	0.47	0.27	0.82	6.03 E-03
**SNORD56**	0.5	0.3	0.83	6.08 E-03
**SNORA70F**	0.4	0.24	0.66	2.41 E-04
**SNORD116-18**	2.49	1.46	4.27	5.64 E-04
**SNORA74A**	2.37	1.4	4	9.27 E-04

**Table 5:** Multivariate analysis comparing the 2-snoRNAs risk model with prognostic variables in CLLs series

**Variable**	**HR**	**Lo 95% CI**	**Up 95% CI**	***p*-val**
**2-snoRNAs MODEL**	2.53	1.48	4.32	0.0007
**UM-CLL**	1.86	0.92	3.79	0.0854
**ZAP-70**	1.27	0.69	2.36	0.4411
**CD38**	2.61	1.43	4.77	0.0018
**del11**	1.76	0.81	3.83	0.1518
**del17**	1.41	0.32	6.31	0.6494
**12+**	1.05	0.52	2.15	0.8867

Multivariate Cox regression analysis was finally performed to test the independence of the 2-snoRNAs risk model from known predictive factors in CLL (*IGHV* mutational status, ZAP-70 and CD38 expression and unfavorable chromosomal aberration) as covariates. The analysis ascertained the independent prognostic value of the 2-snoRNAs model in our series (*p* = 0.0007, Table 5).

## Discussion

The relevance of non-coding RNA sequences in human disease has increased remarkably over the past years. miRNAs represent the most extensively investigated category of ncRNAs in cancer since there are genetic and epigenetic defects causing their deregulation and contributing to tumorigenesis. However, other ncRNAs, such as PIWI-interacting RNAs (piRNAs), small nucleolar RNAs (snoRNAs), transcribed ultraconserved regions (T-UCRs) and large intergenic non-coding RNAs (lincRNAs) are emerging as key elements of cellular homeostasis. The deregulation of these RNAs may contribute to the development of many different human disorders, including cancer [14].

snoRNAs and scaRNAs play important roles in the maturation of rRNA, tRNA, snRNA as well as in mRNA biogenesis [34]. sno/scaRNAs may also be involved in human cancers as demonstrated by recent studies in MM, lymphomas and leukemias [17-22]. In this report we describe for the first time the global expression profiles of sno/scaRNAs in the cells from a large cohort of CLL cases already characterized for many features. These profiles were also compared with those of various sub-populations of normal B-cells from peripheral blood and from tonsils.

The identification of the normal B-cell counterpart of CLL (namely SM and MZ B-cells on one side and N B-cells on the other) is currently a debated question [35,36]. Although based on a limited number of normal samples, our analysis revealed that the sno/scaRNA expression profile of leukemic cells is much more similar to N, MZ and M B-cells than to GC or total pB- cells. Therefore, sno/scaRNAs that significantly discriminate between CLL cells and N, MZ and SM B-cells, might be of relevance in disease pathogenesis. These are SNORA31, -6, -62, and 71C, all of them representing canonical “H/ACA snoRNAs”, which bind to conserved core box H/ACA snoRNP proteins, such as DKC1, GAR1, NHP2 and NOP10, and act mainly as guide RNAs for the site-specific pseudouridylation of specific target rRNA [15]. Notably, all of these ncRNAs were down-regulated in CLL cells (Figure 1B) irrespective of the presence or absence of other genetic lesions (data not shown). Specifically, SNORA31 was significantly down-regulated in association with its host gene *TPT1* (Table 3), which encodes for a critical protein involved in the control of stemness, pluripotency, or tumor reversion [37]. In addition, *TPT1* mRNA is capable of activating the dsRNA-dependent protein kinase PKR, which is in turn critical for the tumor suppressor function of TP53 [37]. At present it is unknown whether SNORA31 down-regulation may have a pathological role *per se*, or whether this down-regulation merely represents a marker of *TPT1* down-regulation. SNORA6 and −62, were both down-regulated in association with their common host gene *RPSA* (Table 3). This gene encodes for a protein that, depending on its folding configuration, may have different cellular activities in specific compartments, that is the ribosomal protein SA or the laminin membrane receptor. Cytometric studies reported that the immature laminin receptor which, unlike the mature receptor, is not acylated, is specifically expressed on the surface of CLL B-cells but not in N, GC, or SM B-lymphocytes from normal tonsils. Moreover, its expression correlated with mutated *IGHV* status predicting a favorable prognosis in CLL [38]. These data are in apparent conflict with ours, although it is possible that down-regulation of the *RPSA* gene does not affect the translated gene fraction folded as immature laminin receptor, but rather the co-regulated expression of SNORA6 and −62. Finally, CLL cells significantly down-regulated SNORA71C located in a cluster at chromosome 20q11 including SNORA71A, -71B, and -71D.

Our data based on unsupervised analyses suggest an heterogeneous pattern of sno/scaRNA expression profiles within the major CLL subgroups classified according to biological, molecular and cytogenetic markers. This is different from the evidence in MM showing specific sno/scaRNA expression patterns associated with distinct molecular subgroups of the disease [19]. However, supervised analyses demonstrated specific, albeit limited, signatures of sno/scaRNA expression in distinct molecular and genetic CLL groups (Table 2). To note, SNORA70F was found consistently down-regulated in patients associated with adverse prognostic markers, such as unmutated IGHV, ZAP70 and CD38 positivity, 12+, and del11. SNORA70F is located within the first intron of the *COBLL1* gene, the expression of which correlates significantly with that of SNORA70F in our dataset (Table 3). *COBLL1* is down-regulated in UM-CLLs [39] as well as in CLL with a poor prognosis in general [40]. The exact function of the COBLL1-encoded protein is still unknown, although data have suggested an involvement in mid-brain neural tube closure [41]. Moreover, *COBLL1* is a negative regulator of apoptosis in malignant mesothelioma cells and its expression is associated with a relatively better prognosis [42]. Finally, we provided evidence that patients with del11 or 12+ deregulated sno/scaRNAs located in the respective altered chromosome; in particular, 12+ samples exhibited a significant up-regulation of snoRNAs host genes as well (Table 2), supporting the notion that the gene dosage effects brought about by this lesion may have a pathogenetic value [43].

With regards to the potential clinical relevance of this specific ncRNA family in cancer, recent reports indicated the association of SNORD25, SNORD27, SNORD30, and SNORD31 with progression from smoldering to symptomatic MM [18] and the over-expression of SNORD71 in peripheral T-cell Lymphoma with favorable outcome [22]. In our study, we generated a 2-snoRNAs risk model which appears to be able to distinguish two different prognostic groups in our series of Binet stage A CLL patients, independently of all the other known molecular markers. Specifically, the high-risk group was characterized by the high expression of at least one of the two snoRNAs, SNORA74A and SNORD116-18.

Little is known about SNORA74A, which is found to be processed from the second intron of a long pre-mRNA-like transcript, termed precursor U19H RNA. At least five transcripts are obtained by alternative splicing of the long *U19H* gene, and only one form (approximately 2%) encodes for a protein, MATR3, involved in mRNA stabilization [44,45]. This evidence may explain why SNORA74A expression does not correlate with that of *MATR3*, a phenomenon also observed in MM patients [19]. SNORD116-18 belongs to a cluster of 29 highly similar copies that, together with the SNORD115 cluster, are tandemly-arranged within the introns of the SNURF–SNRPN transcript, which contains at least 148 exons spanning more than 460 kb on chromosome 15q11. This genomic region is known to be affected by minimal deletions associated with the Prader–Willi syndrome (PWS) [46]. The expression of *SNURF–SNRPN*, as well as that of other several genes in this region, is finely regulated by a bipartite imprinting center (IC) which silences most maternal genes of the PWS critical region allowing the *SNURF–SNRPN* pre-mRNA to be expressed only from the paternal allele. However, mechanisms other than IC methylation may contribute to the deregulated expression of SNORD116 in MM [19]. To note, SNORD116-18 belongs to a subgroup of so-called “orphan” sno/scaRNAs which lack apparent complementarities to cellular RNAs. These molecules may play a role in the regulation of alternative splicing mRNAs, as demonstrated for murine SNORD115 which is processed to a shorter form binding to complementary target sequences on the *HTR2C* pre-mRNA for the correct alternative splicing of the serotonin receptor mRNA [47]. A role in the 3′ processing of selected snoRNAs has also been proposed for SCARNA22 which is deregulated in MM in association with its host gene *MMSET,* involved in the t(4,14) chromosomal translocation [17]. A similar function could be suggested for SNORD116 variants, as also predicted by the recent available snoTARGET open source [48]. In this perspective, it is conceivable that SNORD116-18 deregulation might also have a role in CLL cell since the involvement of uncorrected splicing mechanisms in CLL pathology is supported by increasing evidence [40,49]. Unfortunately, the lack of either independent publicly available datasets or some standardizable methodology prevented the identified 2-snoRNA model from being considered a pure classifier; however, our results provided important indication on the possible role of non-coding RNA in the prognosis of CLL, and prompt for further investigations.

## Conclusions

In conclusion, our data extend the current view of ncRNA deregulation in cancer pointing to the potential relevance of the sno/scaRNA family in the context of CLL which may contribute to discover novel putative molecular markers associated with the disease.

## Competing interests

The authors declare that they have no competing interests.

## Authors’ contributions

Contributions: DR analyzed the data, performed Q-RT-PCR, and wrote the manuscript; LM generated gene expression profiling data and analyzed the data; GT and LA performed statistical and integrative analyses; SF and GC performed FISH; SM, CM, MC, MG, SB, and GC performed the characterization of CLL samples for prognostic markers and cell purification; SM, CM, and DR performed B-cell subsets preparation and cell sorting; MN, FM, MF, AGR, and PT critically reviewed the manuscript; AN designed the study and wrote the manuscript. All authors approved the final version of the manuscript.

## Pre-publication history

The pre-publication history for this paper can be accessed here:

http://www.biomedcentral.com/1755-8794/6/27/prepub

## Supplementary Material

Additional file 1 Supplemental Methods. Click here for file

Additional file 2 **CD19+ tonsil B cells were stained for IgD/CD38 expression.** Three different populations were gated to obtain: IgD^brigth^CD38^−^CD27^−^naïve (N) B cells; IgD^−^CD38^+^ (GC, germinal center) and IgD^−/low^CD38^−^CD27^+^ (memory B cells). The latter were further separated into IgM^+^ (MZ, analogous to marginal zone-like B cells) and IgM^−^ (SM, switched memory) B cells. Click here for file

Additional file 3 **Plots of the density distributions of SNORA74A and SNORD116-18 expression across 191 samples.** Green and orange dots distinguish low and high expression whereas the vertical dot lines indicate the thresholds. Click here for file

Additional file 4 **Supervised analysis comparing peripheral B-cell and CLL samples. **List of the 103 differentially expressed sno/scaRNAs identified by a high stringent supervised analysis between peripheral B-cell and CLL patients (SAM, q-value 0). Click here for file

Additional file 5 **List of sno/scaRNA genes varying 1.5-fold from the mean across the dataset.** Sno/scaRNAs are ordered according to the *p*-value obtained with the global test, that measured the association (positive or negative, as indicated) between each of the 80 most variable sno/scaRNAs and Progression Free Survival (PFS). The 5 sno/scaRNAs showing a significant association with PFS (P < .05) were highlighted in pink. Click here for file

Additional file 6 **Unsupervised analysis. Hierarchical agglomerative clustering of the samples using the 80 most variable sno/scaRNAs (see Additional file ****5****, patients in columns, snoRNAs in rows) was performed adopting Pearson and average as distance and linkage methods, respectively.** The color scale bar represents the relative sno/scaRNA expression changes normalized by the standard deviation. The patients’ molecular characteristics are shown above the matrix; *n* indicates unavailable information. Click here for file

Additional file 7 **SNORA70F expression levels in the cohort of 211 CLL samples.** The patients’ molecular characteristics are shown below the histogram; each patients were assigned a score (1–5) according to the number of adverse clinical and molecular characteristic in the same patients. Cumulative adverse characteristic in the same sample (score 5) is associated with the lower SNORA70F expression. Click here for file

Additional file 8 List of the sno/scaRNAs significantly correlating with matching host genes (Kendall corrected q-value < .05). Click here for file

Additional file 9 **Multivariate analysis.** Multivariate Cox regression analysis testing the independence of the 5 snoRNAs significant associated with PFS from known predictive factors in CLL as covariates. Click here for file
