# Supplementary material for: Small nucleolar RNAs as new biomarkers in chronic lymphocytic leukemia
Source: BMC Med Genomics. 2013 Sep 3;6:27. doi: 10.1186/1755-8794-6-27 (PMC3766210; doi:10.1186/1755-8794-6-27)
Supplement: Additional file 1 — Supplemental Methods. [file 1755-8794-6-27-S1.pdf]

Additional file 1.pdf- Supplemental Methods

Peripheral blood mononuclear cells from CLL patients were isolated by Ficoll-Hypaque (Seromed, Biochrom KG, Berlin, Germany) density-gradient centrifugation. For sno/scaRNA expression profiling experiments, CLL cells were enriched by negative selection with the EasySep-Human B-cell enrichment kit without CD43 depletion (Stem Cell Technologies, Voden Medical Instruments spa, Milan, Italy) using the fully automated protocol of immunomagnetic cell separation with RoboSep™ (Stem Cell Technologies). The proportion of CD5/CD19/CD23 triple positive B-cells in the suspension was determined by direct immunofluorescence with mABs to CD19- FITC (BD Biosciences Pharmigen, San Josè California, USA), CD23 -PE (BD Biosciences), and CD5- PC5 (Beckman Coulter Immunotech, Marseille, France).

Normal B-lymphocytes were obtained from either buffy coats or tonsils. Tonsil samples were first fine-minced in normal culture medium (RPMI1640, 10% FBS, Life Technologies, Paisley, UK) and passed through a cell strainer (BD) with a 70 µm grid to obtain single cell suspensions. Total B-cells were first enriched by using EasySep human B-cell enrichment kit, negative selection (Stem cell Technology, Vancouver, Canada) using RoboSep instrument (Stemcell Technology). B-cells from buffy coats were further purified by FACS sorting (FACS Aria II, BD, San Jose, CA, USA) using anti-CD19 APC (BD) to obtain purified CD19+ B-cells. Tonsil B-cells were first stained with the following antibodies: FITC-polyclonal anti-IgD (Dako, Glostrup, Denmark), APC anti-CD19 mAb (BD), PE-polyclonal anti-IgM (Dako), Pe-Cy5 (Beckman Coulter, Brea, CA, USA) or PE-CF594 (BD) anti-CD27 mAb, PE-Cy7 anti-CD38 mAb (BD). Gating CD19+ B-cells were further purified by FACS sorting based on the expression of IgD versus CD38. In particular, the following non abutting gates were drawn to separate naïve B-cells (N) ($IgD^{bright}CD38^{-}CD27^{-}$); marginal zone (MZ)-like B-cells

(from now on word MZ) ($IgD^{low}IgM^{bright}$ $CD38^{-}$); germinal center (GC) B cells ($IgD^{-}CD38^{+}$) and switched memory (SM) B cells ($IgD^{-}IgM^{-}CD38^{-}$) (see Figure S1).
