# Supplementary figures and images for: Small nucleolar RNAs as new biomarkers in chronic lymphocytic leukemia
Source: BMC Med Genomics. 2013 Sep 3;6:27. doi: 10.1186/1755-8794-6-27 (PMC3766210; doi:10.1186/1755-8794-6-27)

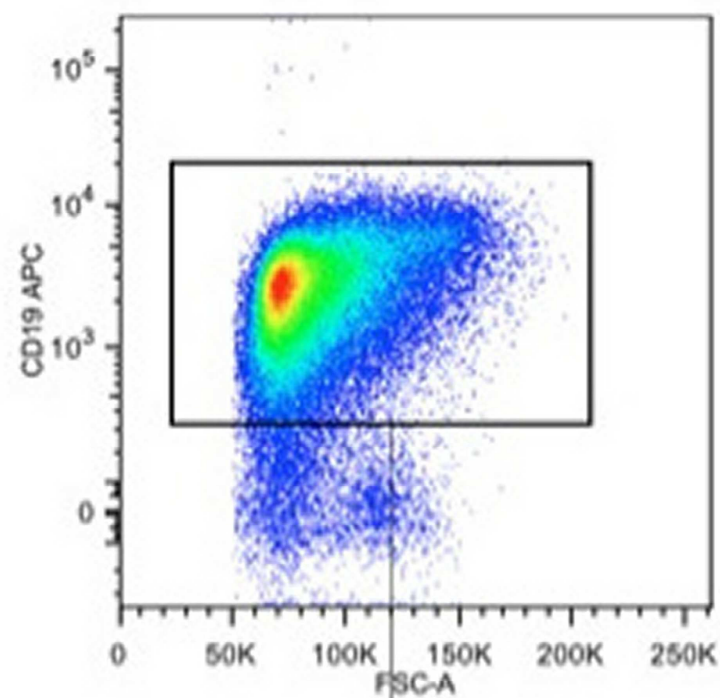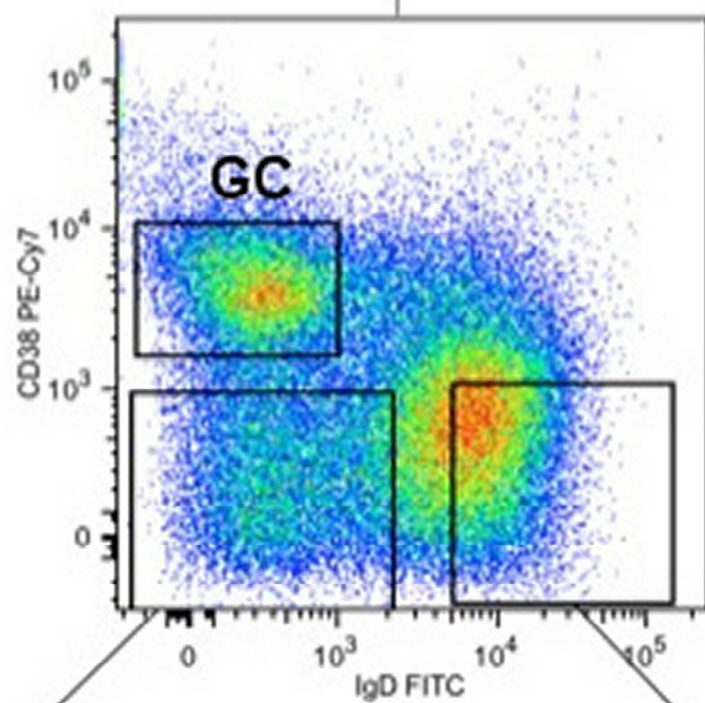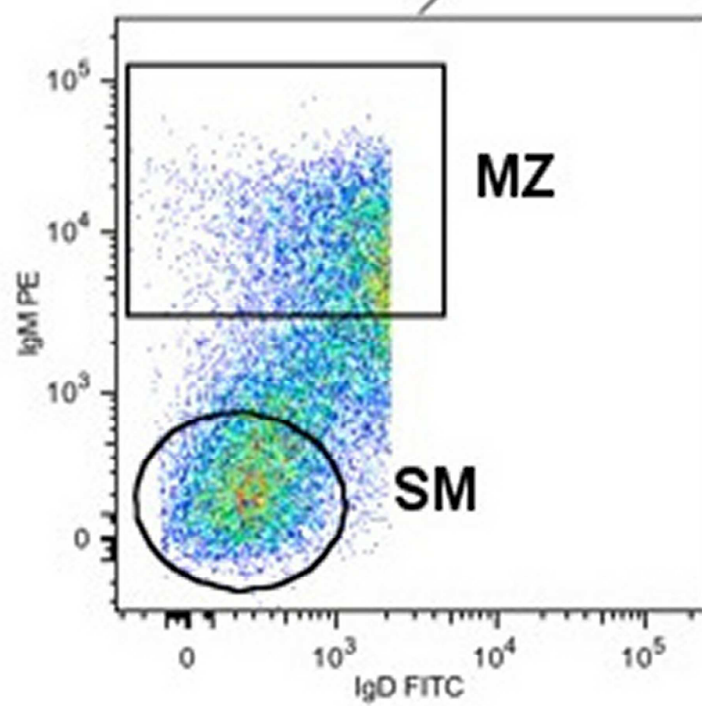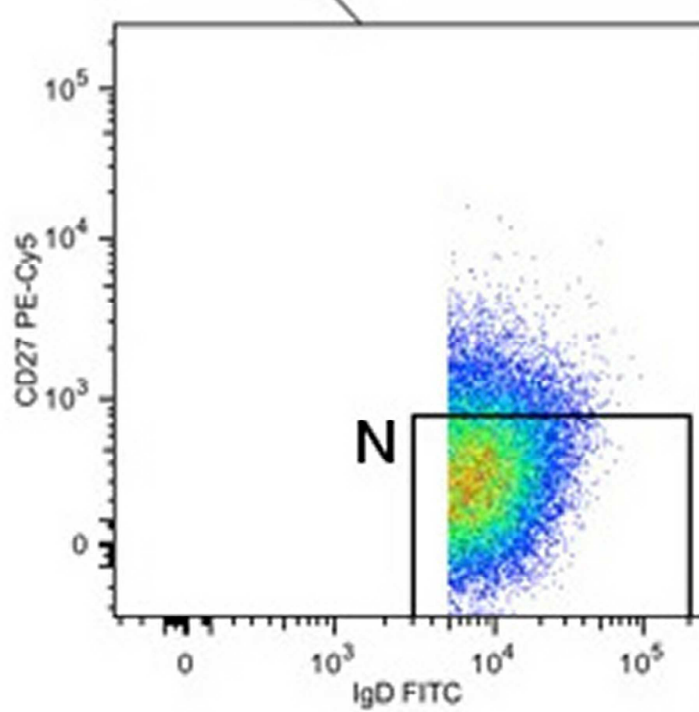

Supplement: Additional file 2 — CD19+ tonsil B cells were stained for IgD/CD38 expression. Three different populations were gated to obtain: IgDbrigthCD38−CD27−naïve (N) B cells; IgD−CD38+ (GC, germinal center) and IgD−/lowCD38−CD27+ (memory B cells). The latter were further separated into IgM+ (MZ, analogous to marginal zone-like B cells) and IgM− (SM, switched memory) B cells. [file 1755-8794-6-27-S2.pdf]

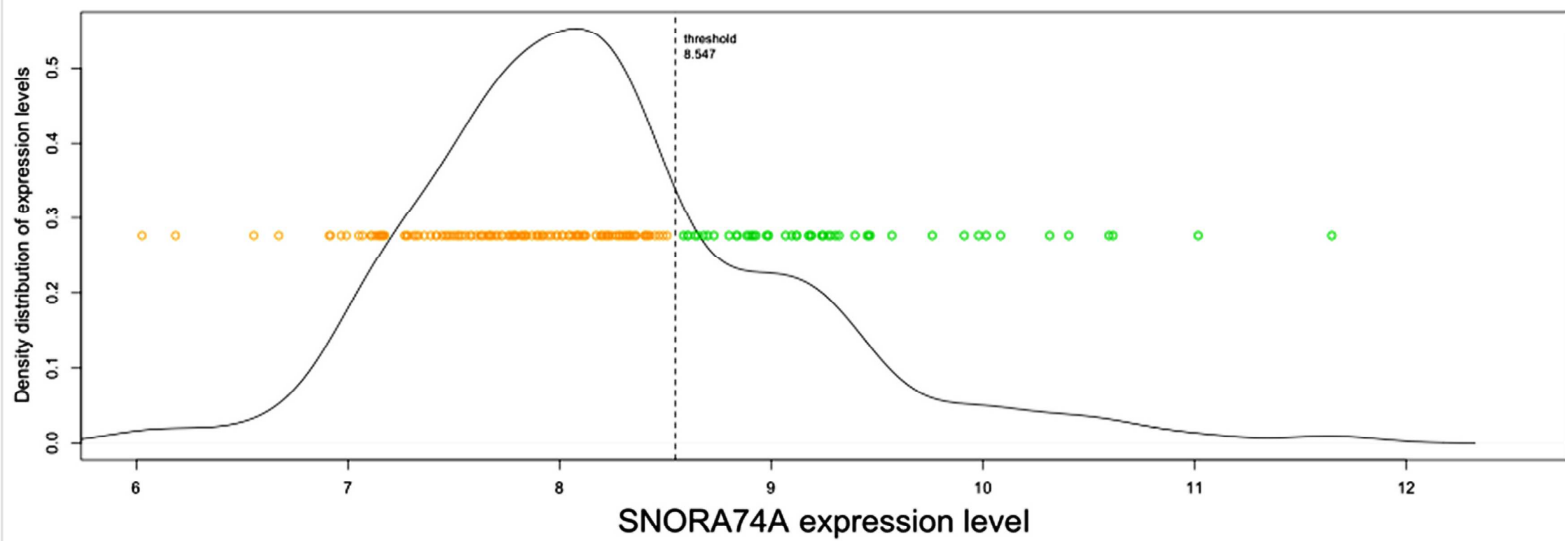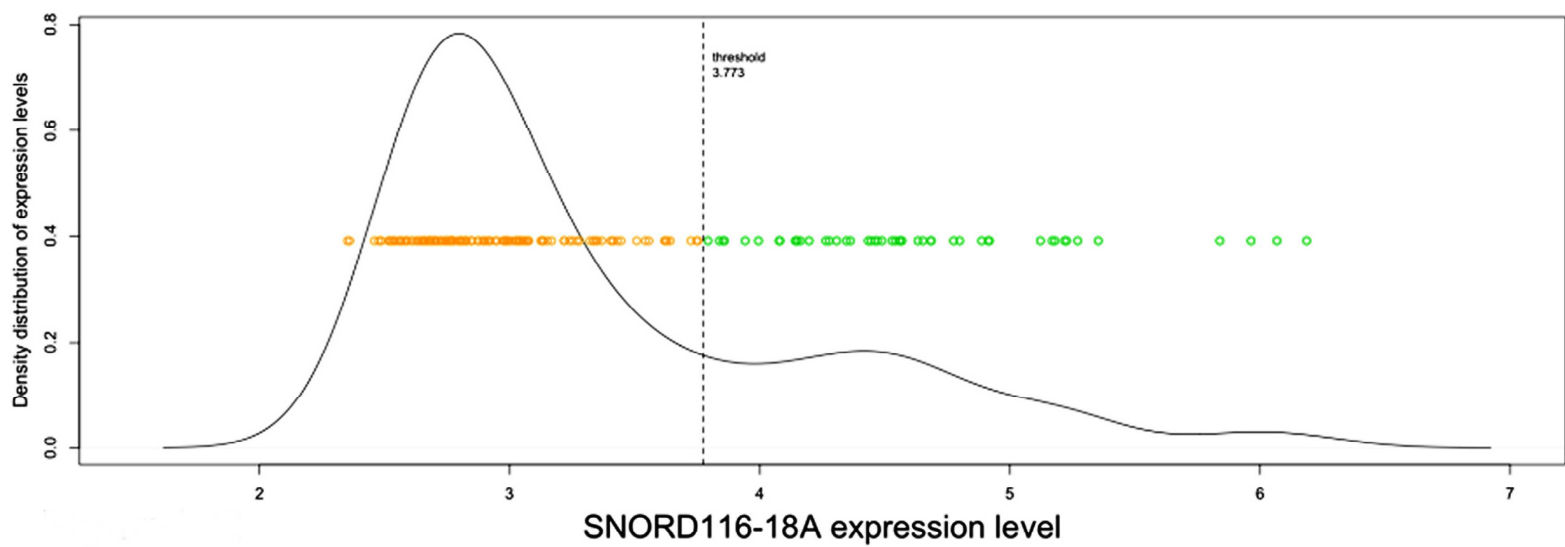

Supplement: Additional file 3 — Plots of the density distributions of SNORA74A and SNORD116-18 expression across 191 samples. Green and orange dots distinguish low and high expression whereas the vertical dot lines indicate the thresholds. [file 1755-8794-6-27-S3.pdf]

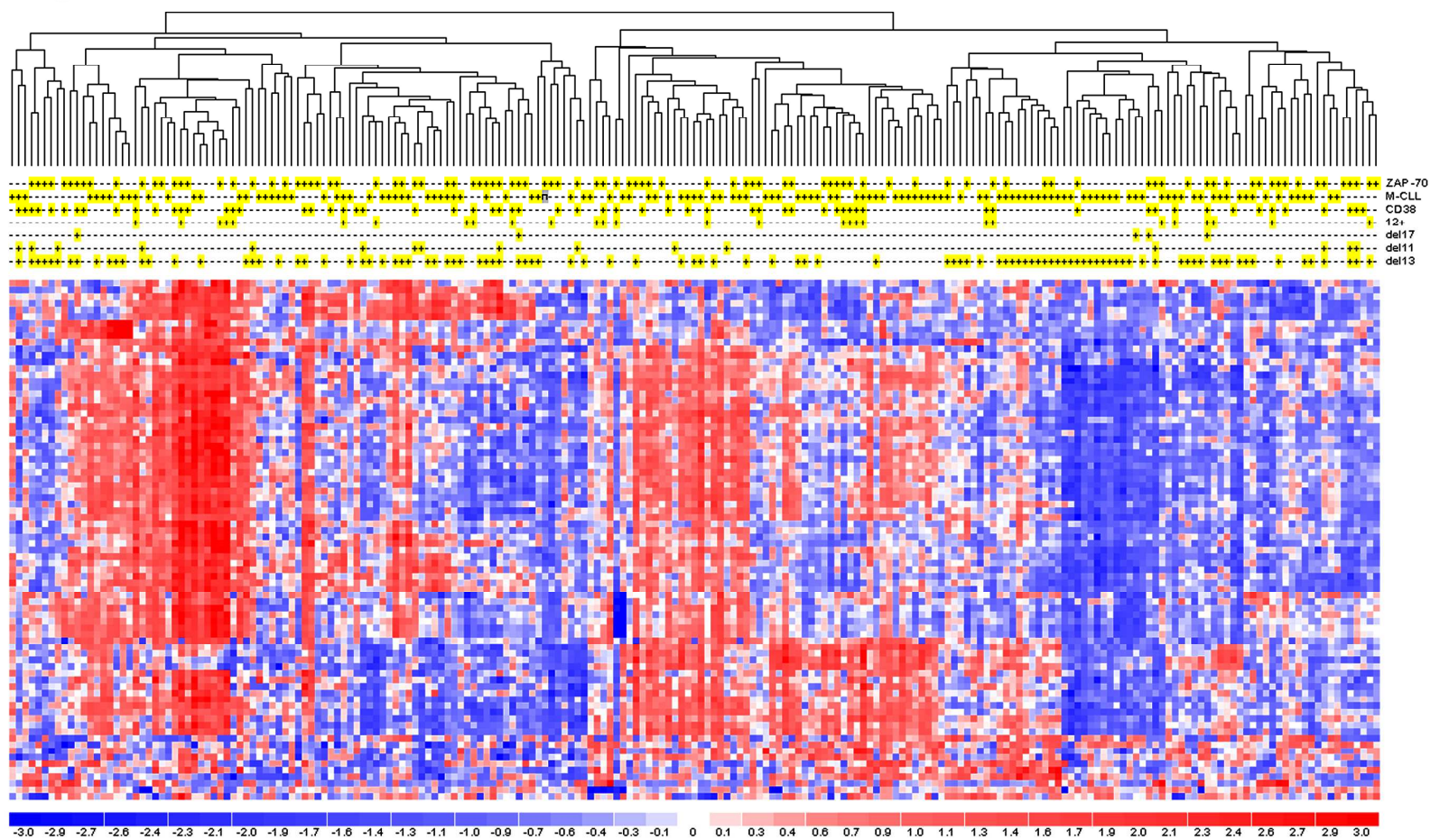

Supplement: Additional file 6 — Unsupervised analysis. Hierarchical agglomerative clustering of the samples using the 80 most variable sno/scaRNAs (see Additional file 5, patients in columns, snoRNAs in rows) was performed adopting Pearson and average as distance and linkage methods, respectively. The color scale bar represents the relative sno/scaRNA expression changes normalized by the standard deviation. The patients’ molecular characteristics are shown above the matrix; n indicates unavailable information. [file 1755-8794-6-27-S6.pdf]

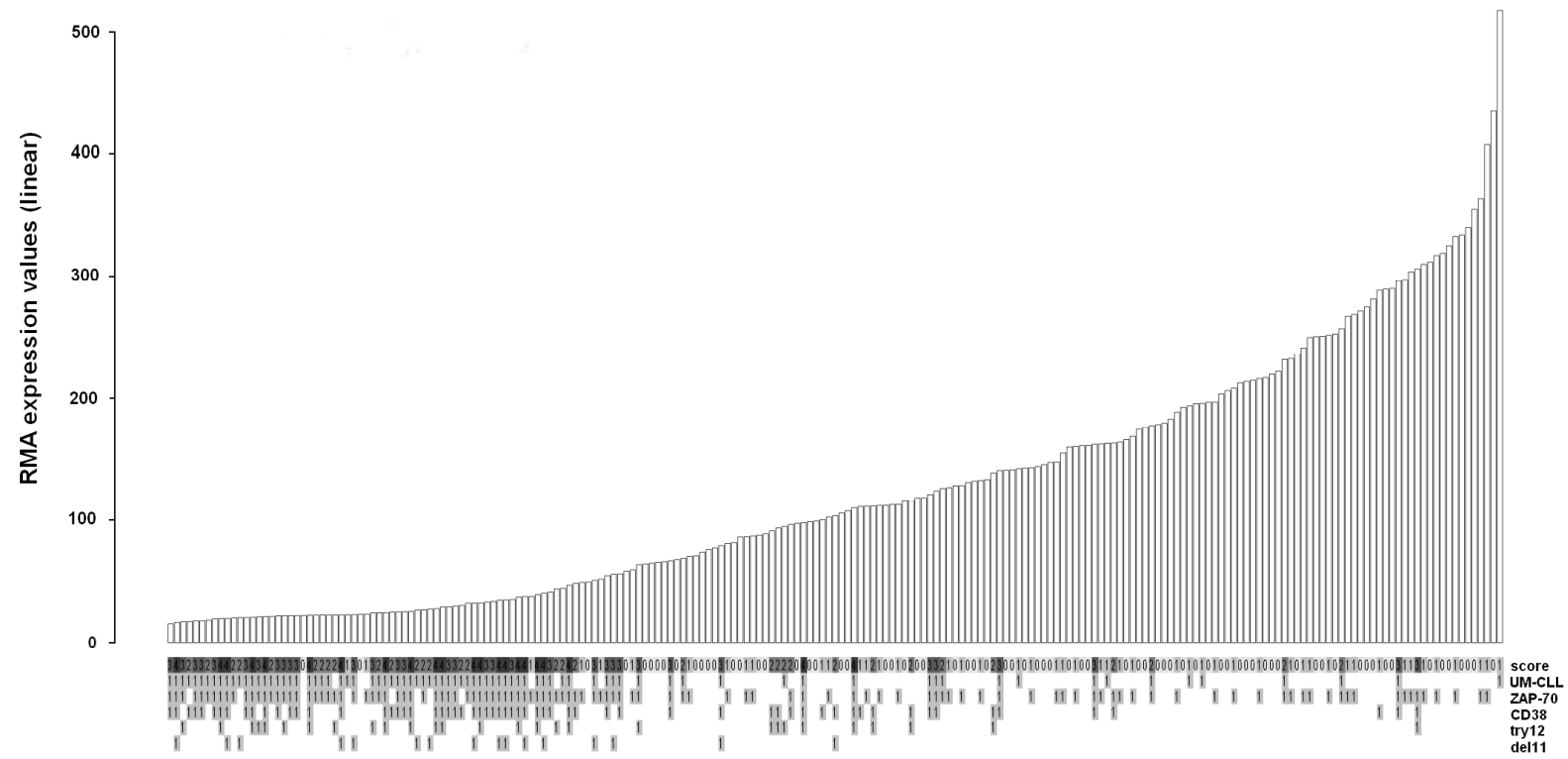

Supplement: Additional file 7 — SNORA70F expression levels in the cohort of 211 CLL samples. The patients’ molecular characteristics are shown below the histogram; each patients were assigned a score (1–5) according to the number of adverse clinical and molecular characteristic in the same patients. Cumulative adverse characteristic in the same sample (score 5) is associated with the lower SNORA70F expression. [file 1755-8794-6-27-S7.pdf]
