# Supplementary material for: Small nucleolar RNAs as new biomarkers in chronic lymphocytic leukemia
Source: BMC Med Genomics. 2013 Sep 3;6:27. doi: 10.1186/1755-8794-6-27 (PMC3766210; doi:10.1186/1755-8794-6-27)
Supplement: Additional file 4 — Supervised analysis comparing peripheral B-cell and CLL samples. List of the 103 differentially expressed sno/scaRNAs identified by a high stringent supervised analysis between peripheral B-cell and CLL patients (SAM, q-value 0). [file 1755-8794-6-27-S4.pdf]

Additional file 4 - Supervised analysis comparing peripheral B-cell and CLL samples. List of the 103 differentially expressed sno/scaRNAs identified by a high stringent supervised analysis between peripheral B-cell and CLL patients (SAM, q-value 0).

sno/scaRNA	alias	cytoband	score(d)	Fold Change
**SNORD28**	U28	11q12.3	8.88	4.28
**SNORD27**	U27	11q12.3	8.56	5.19
**SNORD30**	U30	11q12.3	8.39	4.53
**SNORD26**	U26	11q12.3	8.36	3.42
**SNORD22**	U22	11q13	7.98	3.66
**SNORD31**	U31	11q12.3	7.53	3.28
**SNORD81**	U81	1q25.1	6.79	3.81
**SNORD50A**	U50	6q14.3	6.65	5.65
**SNORD59B**	U59B	12q13.3	6.57	3.58
**SNORD59A**	U59A	12p13.31	6.29	3.21
**SNORD50B**	U50B	6q14.3	6.27	5.04
**SCARNA9**	mgU2-19/30	11q21	6.23	3.64
**SNORD61**	U61	Xq26.3	6.09	3.86
**SNORD47**	U47	1q25.1	5.91	3.39
**SNORD54**	U54	8q12.1	5.84	3.86
**SNORD57**	U57	20p13	5.84	1.69
**SNORA70D**	U70D	16q22	5.81	1.73
**SNORA54**	ACA54	11p15.4	5.81	3.60
**SNORA27**	ACA27	13q12.2	5.73	2.19
**SNORD21**	U21	1p22.1	5.67	2.77
**SCARNA7**	U90	3q25.22	5.66	2.72
**SNORD44**	U44	1q25.1	5.65	3.51
**SNORA68**	U68	19p13.11	5.63	2.96
**SNORD102**	U102	13q12.2	5.59	2.81
**SNORA28**	ACA28	14q32.32	5.58	3.27
**SNORA75**	U23	2q37.1	5.57	2.22
**SNORA7B**	ACA7B	3q21.3	5.51	3.48
**SNORD116-4**	HBII-85-4	15q11.2	5.51	3.09
**SNORD82**	U82	2q37.1	5.42	3.21
**SNORA33**	ACA33	6q23.2	5.41	3.44
**SNORD116-25**	HBII-85-25	15q11.2	5.40	4.80
**SNORD116-29**	HBII-85-29	15q11.2	5.40	4.10
**SNORD76**	U76	1q25.1	5.39	3.09
**SNORD32A**	U32A	19q13.33	5.39	1.88
**SNORD34**	U34	19q13.33	5.37	3.23
**SNORD12C**	U106	20q13.13	5.36	2.64
**SCARNA9L**	SCARNA9L	Xp22.12	5.35	3.15
**SNORA56**	ACA56	Xq28	5.30	2.09
**SNORD33**	U33	19q13.33	5.28	1.60
**SNORD51**	U51	2q33.3	5.28	2.75
**SNORD60**	U60	16p13.3	5.27	5.64

**SNORD52**	U52	6p21.33	5.27	3.39
**SNORA6**	ACA6	3p22.2	5.23	2.50
**SNORD14C**	SNORD14C	11q24.1	5.22	2.19
**SNORD55**	U55	1p34.1	5.18	3.31
**SNORA70E**	U70E	11q14	5.17	2.81
**SNORA29**	ACA29	6q25.3	5.11	2.42
**SNORD37**	U37	19p13.3	5.10	2.89
**SNORA19**	ACA19	10q26.11	5.05	2.96
**SCARNA6**	U88	2q37.1	4.92	2.86
**SNORD78**	U78	1q25.1	4.88	3.09
**SNORD25**	U25	11q13	4.86	2.63
**SNORD79**	U79	1q25.1	4.85	2.57
**SNORD116-26**	HBII-85-26	15q11.2	4.83	4.08
**SNORD105**	U105	19p13.2	4.82	3.29
**SNORD94**	U94	2p11.2	4.81	2.46
**SNORD63**	U63	5q31.2	4.80	3.57
**SNORA38**	ACA38	6p21.33	4.79	1.93
**SNORD74**	U74	1q25.1	4.78	1.85
**SNORD4A**	mgh18S-121	17q11	4.75	2.07
**SNORD46**	U46	1p34.1	4.74	2.66
**SNORD68**	HBII-202	16q24.3	4.71	2.07
**SNORD116-1**	HBII-85-1	15q11.2	4.70	2.27
**SNORD80**	U80	1q25.1	4.69	2.14
**SCARNA5**	U87	2q37.1	4.65	2.70
**SNORA20**	ACA20	6q25.3	4.64	2.77
**SCARNA17**	U91	18q21.1	4.62	2.35
**SNORD75**	U75	1q25.1	4.59	2.82
**SNORA2B**	ACA2B	12q13.11	4.59	2.41
**SNORD58A**	U58A	18q21.1	4.58	2.49
**SNORA64**	U64	16p13.3	4.52	2.80
**SNORA2A**	ACA2A	12q13.11	4.51	2.48
**SNORA16A**	ACA16	1p35.3	4.49	3.59
**SNORA62**	E2	3p22.2	4.47	2.29
**SNORD8**	mgU6-53	14q11.2	4.46	2.82
**SNORD116-11**	HBII-85-11	15q11.2	4.45	3.02
**SNORD116-13**	HBII-85-13	15q11.2	4.42	3.69
**SNORD35A**	U35A	19q13.33	4.42	2.13
**SNORD95**	U95	5q35.3	4.40	2.43
**SNORD24**	U24	9q34.2	4.37	2.97
**SNORD14E**	SNORD14E	11q24.1	4.36	3.03
**SNORA69**	U69	Xq24	4.28	2.25
**SNORD116-28**	HBII-85-28	15q11.2	4.28	2.06
**SNORA44**	ACA44	1p35.3	4.20	2.02
**SNORD48**	U48	6p21.33	4.19	2.42
**SNORA24**	ACA24	4q26	4.19	3.23
**SNORA74A**	U19	5q31.2	4.14	3.10
**SNORA80**	ACA67	21q22.11	4.14	1.54

**SNORA50**	ACA50	16q21	4.13	1.77
**SCARNA11**	ACA57	12p13.31	4.12	1.77
**SNORA45**	ACA3-2	11p15.4	4.12	2.37
**SNORD42B**	U42B	17q11.2	4.11	2.01
**SNORD116-27**	HBII-85-27	15q11.2	4.09	2.18
**SNORD56**	U56	20p13	4.08	2.49
**SNORD116-22**	HBII-85-22	15q11.2	4.05	2.34
**SNORD116-16**	HBII-85-16	15q11.2	4.04	2.58
**SNORD104**	U104	17q23.3	4.02	2.28
**SNORA4**	ACA4	3q27.3	4.00	1.97
**SNORD43**	U43	22q13.1	3.99	2.26
**SNORD73A**	U73a	4q31.3	3.97	1.97
**SNORA46**	ACA46	16q21	3.97	2.80
**SNORA49**	ACA49	12q24.33	3.93	2.19
**SNORA36C**	SNORA36C	2p14	-7.66	0.40
