# Supplementary material for: Small nucleolar RNAs as new biomarkers in chronic lymphocytic leukemia
Source: BMC Med Genomics. 2013 Sep 3;6:27. doi: 10.1186/1755-8794-6-27 (PMC3766210; doi:10.1186/1755-8794-6-27)
Supplement: Additional file 5 — List of sno/scaRNA genes varying 1.5-fold from the mean across the dataset. Sno/scaRNAs are ordered according to the p-value obtained with the global test, that measured the association (positive or negative, as indicated) between each of the 80 most variable sno/scaRNAs and Progression Free Survival (PFS). The 5 sno/scaRNAs showing a significant association with PFS (P < .05) were highlighted in pink. [file 1755-8794-6-27-S5.pdf]

Additional file 5.pdf- List of sno/scaRNA genes varying 1.5-fold from the mean across the dataset. Sno/scaRNAs are ordered according to the *p*-value obtained with the global test, that measured the association (positive or negative, as indicated) between each of the 80 most variable sno/scaRNAs and Progression Free Survival (PFS). The 5 sno/scaRNAs showing a significant association with PFS (P<.05) were highlighted in pink.

sno/scaRNA	alias	cytoband	Fold Change	association with PFS	Global Test *p*-value
**SNORA70F**	U70F	2q24	2.71	+	5.50E-05
**SNORD116-18**	HBII-85-18	15q11.2	1.57	-	2.48E-04
**SNORD1A**	snR38A	17q25.1	1.57	+	2.47E-03
**SNORA74A**	U19	5q31.2	1.82	-	1.02E-02
**SNORD56**	U56	20p13	1.55	+	3.17E-02
**SCARNA4**	ACA26	1q22	2.15	-	5.83E-02
**SNORD54**	U54	8q12.1	1.59	+	6.04E-02
**SCARNA22**	ACA11	4p16.3	1.59	-	7.78E-02
**SNORD78**	U78	1q25.1	1.59	+	8.48E-02
**SNORD56B**	-	14q24.2	1.76	+	8.87E-02
**SCARNA10**	U85	12p13	1.57	-	1.08E-01
**SNORD115-25**	HBII-52-25	15q11.2	1.71	+	1.50E-01
**SNORD116-13**	HBII-85-13	15q11.2	1.93	-	2.26E-01
**SNORA19**	ACA19	10q26.11	1.52	+	2.97E-01
**SNORD45B**	U45B	1p31.1	2.80	+	3.25E-01
**SCARNA6**	U88	2q37	1.52	-	3.33E-01
**SNORA52**	ACA52	11p15.5	1.65	-	3.41E-01
**SNORD82**	U82	2q37.1	1.53	-	3.48E-01
**SCARNA1**	ACA35	1p35	1.60	-	3.67E-01
**SNORA14A**	ACA14A	7q11.23	1.91	-	3.69E-01
**SNORA38B**	-	17q24.2	2.25	-	3.83E-01
**SNORD116-11**	HBII-85-11	15q11.2	1.68	-	4.19E-01
**SNORA16A**	ACA16	1p35.3	1.84	+	4.20E-01
**SNORA21**	ACA21	17q12	1.68	-	4.28E-01
**SNORA60**	ACA60	20q11.23	1.94	-	4.39E-01
**SNORA7B**	ACA7B	3q21.3	1.58	-	4.61E-01
**SNORA14B**	ACA14B	1q42.3	1.63	-	4.71E-01
**SNORD52**	U52	6p21.33	1.60	+	4.89E-01
**SNORD44**	U44	1q25.1	1.55	+	4.90E-01
**SNORD38B**	U38B	1p34.1	1.87	+	5.08E-01
**SNORA41**	ACA41	2q33.3	1.77	-	5.14E-01
**SNORD1C**	snR38C	17q25.1	1.61	-	5.21E-01
**SNORD116-6**	HBII-85-6	15q11.2	1.50	-	5.35E-01
**SNORA57**	U99	11q12.2	1.58	-	5.45E-01
**SNORA42**	ACA42	1q22	1.54	-	5.52E-01
**SNORA33**	ACA33	6q23.2	1.57	+	5.97E-01
**SCARNA5**	U87	2q37	1.53	-	6.07E-01
**SNORD77**	U77	1q25.1	1.69	+	6.21E-01

**SNORA65**	U65	9q33.3	1.94	-	6.33E-01
**SNORA20**	ACA20	6q25.3	1.52	+	6.38E-01
**SNORA36A**	ACA36	Xq28	2.09	-	6.43E-01
**SNORD116-29**	HBII-85-29	15q11.2	1.76	-	6.48E-01
**SNORA45**	ACA3-2	11p15.4	1.51	+	6.48E-01
**SNORD116-26**	HBII-85-26	15q11.2	1.93	-	6.55E-01
**SNORD24**	U24	9q34.2	1.67	-	6.56E-01
**SNORD116-25**	HBII-85-25	15q11.2	1.88	-	6.73E-01
**SCARNA8**	U92	9p22.1	1.51	-	6.82E-01
**SNORD61**	U61	Xq26.3	1.57	+	6.83E-01
**SNORD50A**	U50	6q14.3	1.74	+	7.01E-01
**SNORD105**	U105	19p13.2	1.67	-	7.05E-01
**SNORD116-16**	HBII-85-16	15q11.2	1.60	-	7.13E-01
**SNORD50B**	U50B	6q14.3	1.72	+	7.35E-01
**SNORD116-21**	HBII-85-21	15q11.2	2.21	+	7.38E-01
**SNORA64**	U64	16p13.3	1.59	-	7.42E-01
**SNORA37**	ACA37	18q21.2	1.55	-	7.45E-01
**SNORD55**	U55	1p34.1	1.60	-	7.56E-01
**SCARNA23**	ACA12	Xp22.11	1.75	-	7.63E-01
**SNORA13**	ACA13	5q22.2	1.65	-	7.74E-01
**SNORD34**	U34	19q13.33	1.55	+	7.75E-01
**SNORD63**	U63	5q31.2	1.77	+	7.89E-01
**SNORD115-32**	HBII-52-32	15q11.2	1.99	+	7.93E-01
**SNORD6**	mgh28S-2412	11q21	1.70	-	7.98E-01
**SNORA54**	ACA54	11p15.4	1.57	-	8.04E-01
**SNORD15B**	U15B	11q13.4	1.61	-	8.15E-01
**SNORD60**	U60	16p13.3	2.15	-	8.19E-01
**SNORA28**	ACA28	14q32.32	1.52	+	8.25E-01
**SNORA40**	ACA40	11q21	1.60	+	8.36E-01
**SNORD83A**	U83A	22q13.1	1.50	+	8.40E-01
**SNORD76**	U76	1q25.1	1.52	+	8.43E-01
**SNORA24**	ACA24	4q26	1.81	-	8.43E-01
**SNORD75**	U75	1q25.1	1.58	-	8.80E-01
**SNORD115-31**	HBII-52-31	15q11.2	1.67	+	8.80E-01
**SCARNA9L**	-	Xp22.12	1.51	+	8.82E-01
**SNORD20**	U20	2q37.1	1.63	-	8.85E-01
**SNORD8**	mgU6-53	14q11.2	1.62	+	8.87E-01
**SNORD49B**	U49B	17p11.2	2.10	-	8.97E-01
**SNORD37**	U37	19p13.3	1.50	+	9.07E-01
**SNORA46**	ACA46	16q21	1.75	+	9.27E-01
**SNORD49A**	U49A	17p11.2	1.64	+	9.48E-01
**SNORD14E**	-	11q24.1	1.71	-	9.54E-01
