# Supplementary material for: Small nucleolar RNAs as new biomarkers in chronic lymphocytic leukemia
Source: BMC Med Genomics. 2013 Sep 3;6:27. doi: 10.1186/1755-8794-6-27 (PMC3766210; doi:10.1186/1755-8794-6-27)
Supplement: Additional file 8 — List of the sno/scaRNAs significantly correlating with matching host genes (Kendall corrected q-value < .05). [file 1755-8794-6-27-S8.pdf]

Additional file 8. List of the sno/scaRNAs significantly correlating with matching host genes (Kendall corrected q-value < .05).

sno/scaRNA	HOST GENE	q-value
SNORA2B	C12orf41	< 2.2e-16
SNORA70F	COBLL1	< 2.2e-16
SCARNA9L	EIF1AX	< 2.2e-16
SNORD56	NOP56	< 2.2e-16
SNORA16B	PPP2R5A	< 2.2e-16
SNORA70E	RAB30	< 2.2e-16
SNORA21	RPL23	< 2.2e-16
SNORA33	RPS12	< 2.2e-16
SNORD54	RPS20	< 2.2e-16
SNORD15B	RPS3	< 2.2e-16
SNORA61	SNHG12	< 2.2e-16
SNORD5	TAF1D	< 2.2e-16
SNORA30	SRCAP	1.74E-14
SNORD12C	ZNFX1-AS1	1.74E-14
SNORD45C	RABGGTB	4.65E-14
SNORA58	MRPL3	5.66E-14
SCARNA12	PHB2	7.26E-14
SNORD45B	RABGGTB	7.26E-14
SNORD20	NCL	1.14E-12
SNORA65	RPL12	1.84E-12
SNORA5A	TBRG4	1.37E-11
SNORA44	SNHG12	1.41E-11
SNORA4	EIF4A2	2.92E-11
SNORD101	RPS12	3.48E-11
SNORA70B	USP34	3.68E-11
SNORA1	TAF1D	1.68E-10
SNORA56	DKC1	4.85E-10
SNORA75	NCL	4.85E-10
SNORA13	EPB41L4A-AS1	8.50E-09
SNORA5C	TBRG4	1.36E-08
SNORD52	C6orf48	2.11E-08
SNORA41	EEF1B2	2.39E-08
SNORD15A	RPS3	2.39E-08
SNORD58A	RPL17	2.41E-08
SNORA29	TCP1	5.22E-08
SNORA8	TAF1D	8.79E-08
SNORA5B	TBRG4	9.60E-08
SNORA40	TAF1D	2.96E-07
SNORD59B	ATP5B	3.53E-07
SCARNA11	CHD4	7.12E-07
SNORA54	NAP1L4	7.86E-07
SNORA28	EIF5	8.17E-07
SNORA55	PABPC4	1.24E-06

sno/scaRNA	HOST GENE	q-value
SNORA19	EIF3A	1.89E-06
SNORA2A	C12orf41	2.00E-06
SNORA70C	ASTN2	3.76E-06
SNORA6	RPSA	4.18E-06
SNORA62	RPSA	6.38E-06
SNORD51	EEF1B2	9.80E-06
SNORD82	NCL	1.05E-05
SNORD68	RPL13	1.24E-05
SNORA80B	ODC1	3.12E-05
SNORA51	NOP56	3.89E-05
SNORD14C	HSPA8	0.000
SNORD48	C6orf48	0.000
SNORD14E	HSPA8	0.000
SNORD45A	RABGGTB	0.000
SNORD59A	ATP5B	0.001
SNORD96B	AMMECR1	0.001
SNORA36A	DKC1	0.002
SNORA35	HTR2C	0.002
SNORD21	RPL5	0.002
SNORD37	EEF2	0.002
SNORD61	RBMX	0.004
SNORD105	PPAN	0.005
SNORA60	SNHG11	0.007
SCARNA4	KIAA0907	0.009
SNORD116-10	SNRPN	0.016
SNORD115-40	SNRPN	0.018
SNORD116-15	SNRPN	0.018
SNORA16A	SNHG12	0.025
SNORA36C	AAK1	0.026
SNORA31	TPT1	0.030
SNORD116-23	SNRPN	0.030
SNORD56B	SIPA1L1	0.033
SNORA14A	POR	0.039
SNORA70	RPL10	0.043
SNORA14B	TOMM20	0.043
SNORD63	HSPA9	0.047
