# Supplementary material for: Small nucleolar RNAs as new biomarkers in chronic lymphocytic leukemia
Source: BMC Med Genomics. 2013 Sep 3;6:27. doi: 10.1186/1755-8794-6-27 (PMC3766210; doi:10.1186/1755-8794-6-27)
Supplement: Additional file 9 — Multivariate analysis. Multivariate Cox regression analysis testing the independence of the 5 snoRNAs significant associated with PFS from known predictive factors in CLL as covariates. [file 1755-8794-6-27-S9.pdf]

Additional file 9. Multivariate analysis. Multivariate Cox regression analysis testing the independence of the 5 snoRNAs significant associated with PFS from known predictive factors in CLL as covariates.

VARIABLE	HR	lo95%CI	up95%CI	pval
SNORA74A	2.16	1.27	3.68	0.004
UM-CLL	1.68	0.83	3.42	0.15
ZAP70	1.33	0.73	2.44	0.347
CD38	2.93	1.6	5.34	0.0004
del 11	2.18	1	4.76	0.49
del 17	1.24	0.28	5.52	0.772
12+	1.07	0.52	2.17	0.854

VARIABLE	HR	lo95%CI	up95%CI	pval
SNORA70F	0.96	0.48	1.9	0.905
UM-CLL	1.81	0.82	3.98	0.138
ZAP70	1.31	0.71	2.41	0.388
CD38	2.78	1.53	5.06	0.0008
del 11	2.03	0.93	4.43	0.073
del 17	1.38	0.31	6.06	0.668
12+	1.11	0.54	2.27	0.77

VARIABLE	HR	lo95%CI	up95%CI	pval
SNORD1A	0.58	0.33	1.03	0.06
UM-CLL	1.75	0.88	3.49	0.1
ZAP70	1.36	0.75	2.48	0.3
CD38	2.71	1.5	4.89	0.0009
del 11	1.87	0.86	4.07	0.11
del 17	1.46	0.33	6.36	0.61
12+	1.12	0.55	2.26	0.75

VARIABLE	HR	lo95%CI	up95%CI	pval
SNORD56	0.54	0.32	0.92	0.02
UM-CLL	1.67	0.82	3.41	0.15
ZAP70	1.36	0.74	2.5	0.32
CD38	2.99	1.62	5.52	0.0005
del 11	1.79	0.82	3.9	0.14
del 17	1.35	0.3	5.99	0.69
12+	1.01	0.49	2.06	0.99

VARIABLE	HR	lo95%CI	up95%CI	pval
SNORD116-18	1.82	1	3.19	0.03
UM-CLL	1.81	1	3.64	0.09
ZAP70	1.27	1	2.35	0.44
CD38	2.72	2	4.93	0.001
del 11	1.72	1	3.77	0.17
del 17	1.35	0	5.95	0.69
12+	1.12	1	2.26	0.74
